# Fructose Alters Intermediary Metabolism of Glucose in Human Adipocytes and Diverts Glucose to Serine Oxidation in the One–Carbon Cycle Energy Producing Pathway

**DOI:** 10.3390/metabo5020364

**Published:** 2015-06-16

**Authors:** Vijayalakshmi Varma, László G. Boros, Greg T. Nolen, Ching-Wei Chang, Martin Wabitsch, Richard D. Beger, Jim Kaput

**Affiliations:** 1 Biomarkers and Alternate Models branch, Division of Systems Biology, National Center for Toxicological Research, Jefferson, AR 72079, USA; E-Mails: greg.nolen@fda.hhs.gov (G.T.N.); richard.beger@fda.hhs.gov (R.D.B.); 2 Division of Bioinformatics and Biostatistics, National Center for Toxicological Research, Jefferson, AR 72079, USA; E-Mail: ching-wei.chang@fda.hhs.gov; 3 SiDMAP LLC, Los Angeles, CA 90064, USA; E-Mail: lboros@sidmap.com; 4 Los Angeles Biomedical Research Institute (LABIOMED), Torrance, CA 90502, USA; 5 Department of Pediatrics, Harbor-UCLA Medical Center, Torrance, CA 90502, USA; 6 Division of Pediatric Endocrinology and Diabetology, University of Ulm, Eythstr. 24, 89075 Ulm, Germany; E-Mail: martin.wabitsch@uniklinik-ulm.de; 7 Current Address: Nestle Institute of Health Sciences, 1015 Lausanne, Switzerland; E-Mail: james.kaput@rd.nestle.com

**Keywords:** human adipocytes, fructose, glucose, [1,2-^13^C_2_]-d-glucose, targeted tracer fate association study (TTFAS), SOGC pathway

## Abstract

Increased consumption of sugar and fructose as sweeteners has resulted in the utilization of fructose as an alternative metabolic fuel that may compete with glucose and alter its metabolism. To explore this, human Simpson-Golabi-Behmel Syndrome (SGBS) preadipocytes were differentiated to adipocytes in the presence of 0, 1, 2.5, 5 or 10 mM of fructose added to a medium containing 5 mM of glucose representing the normal blood glucose concentration. Targeted tracer [1,2-^13^C_2_]-d-glucose fate association approach was employed to examine the influence of fructose on the intermediary metabolism of glucose. Increasing concentrations of fructose robustly increased the oxidation of [1,2-^13^C_2_]-d-glucose to ^13^CO_2_ (*p* < 0.000001). However, glucose-derived ^13^CO_2_ negatively correlated with ^13^C labeled glutamate, ^13^C palmitate, and M_+1_ labeled lactate. These are strong markers of limited tricarboxylic acid (TCA) cycle, fatty acid synthesis, pentose cycle fluxes, substrate turnover and NAD^+^/NADP^+^ or ATP production from glucose via complete oxidation, indicating diminished mitochondrial energy metabolism. Contrarily, a positive correlation was observed between glucose-derived ^13^CO_2_ formed and ^13^C oleate and doses of fructose which indicate the elongation and desaturation of palmitate to oleate for storage. Collectively, these results suggest that fructose preferentially drives glucose through serine oxidation glycine cleavage (SOGC pathway) one-carbon cycle for NAD^+^/NADP^+^ production that is utilized in fructose-induced lipogenesis and storage in adipocytes.

## 1. Introduction

Consumption of sugar and fructose as sweeteners with low sodium and fat intake has increased at the same time as nutrition-linked chronic diseases have reached epidemic proportions in humans. The increased consumption of sweetened foods and beverages expose the body to higher amounts of fructose than that naturally occurring in fruits and vegetables. The correlation to chronic disease has led to the implication that excess fructose is involved in the development of insulin resistance and obesity [1,2]. A number of studies have explored the effects of fructose over-consumption in the form of beverages or foods in rodent models and in non-human primates [3,4,5,6].

Fructose can serve as an alternative metabolic fuel in some cells where it may compete with glucose and alter its metabolism [7,8,9]. However, glucose is typically the preferred metabolic fuel for energy production utilized by all the cells of the body and is critical to normal physiological functioning [10,11]. In addition to its important role as a key metabolic fuel, glucose also contributes to increasing the pool of NADPH through its metabolism in the pentose phosphate pathway. NADPH is an important coenzyme used in anabolic reactions such as fatty acid and nucleic acid synthesis [12]. Glucose also serves as (an anaplerotic) substrate for replenishing intermediates of the TCA cycle [13]. Other fuels such as fatty acids, which also produce energy, have been reported to modulate the utilization of glucose. The reciprocal interaction that occurs between glucose and fatty acids fuels is well described as the glucose-fatty acid cycle or Randle’s cycle [14,15]. When fatty acids are present in excess, the Randle cycle exerts an inhibition on the uptake and oxidation of glucose. Similarly, an interaction between the monosaccharides, glucose and fructose, was also recently described in hepatocytes [9]. In those cells, glucose was shown to augment citrate shuttling and promote the efflux of glucose-derived triglycerides from hepatocytes demonstrating the influence of fructose in altering glucose metabolism. An *in vivo* study that examined the effect of acute fructose administration on hepatic glucose metabolism reported both an augmentation of hepatic and extrahepatic insulin resistance in response to fructose [16]. Although the liver is the primary organ to metabolize fructose, skeletal muscle, kidney, and adipose tissue (particularly visceral adipose tissue) are also known to take up and metabolize fructose [17]. The potential exposure of multiple organs to fructose would suggest that this carbohydrate could alter the utilization of glucose in cells other than hepatocytes, particularly adipocytes which play a role in the regulation of energy balance and in glucose homeostasis [18].

A study published in the late 1970s examined the effect of glucose on the metabolism of fructose in rat adipose tissues [19]. However, the impact of fructose on the metabolism of glucose in adipocytes was not examined. In addition, the effect of fructose in human adipocytes has also not yet been explored. Hence, the aim of this study was to examine the influence of fructose on intermediary glucose metabolism and its modulation in human adipocytes. Recent developments in tracer substrate-based metabolomics demonstrated that the *in vitro* applied [1,2-^13^C_2_]-d-glucose tracer is a precise method for characterizing fluxes of the pentose cycle, glycolysis, and the TCA cycle [20]. The use of stable isotopes has been extended to analyze *de novo* (new) fatty acid synthesis, turnover, and modifications via chain shortening or elongation in cell pellets, plasma or tissues [21]. Tracer substrate based metabolomics using ^13^C glucose and deuterium labeling follow roadmap studies as the most efficient and pertinent tools [22] for linking phenotype with specific metabolic processes by the single 1,2 ^13^C_2_-d-glucose tracer’s fate and its associations with fructose dosing. To address the role or influence of fructose on the metabolism of glucose, we designed a tracer substrate-based metabolic study using stable isotope mapping (SIDMAP) methods to characterize intermediary metabolism, product synthesis, and related adaptive events in adipocytes in response to different concentrations of fructose. Targeted single tracer fate association studies internally correct for perturbations that occur during complex substrate exchange reactions (22) that are altered by fructose-induced non-steady state stable isotope labeling mechanisms. This study provides new insights on the fate of ^13^C labeled glucose molecule in the presence of increasing doses of fructose in adipocytes by tracing it through major metabolic pathways.

## 2. Results

### 2.1. Fructose Dose-Dependently Increased the Oxidation of Glucose in Adipocytes

**Figure 1:**
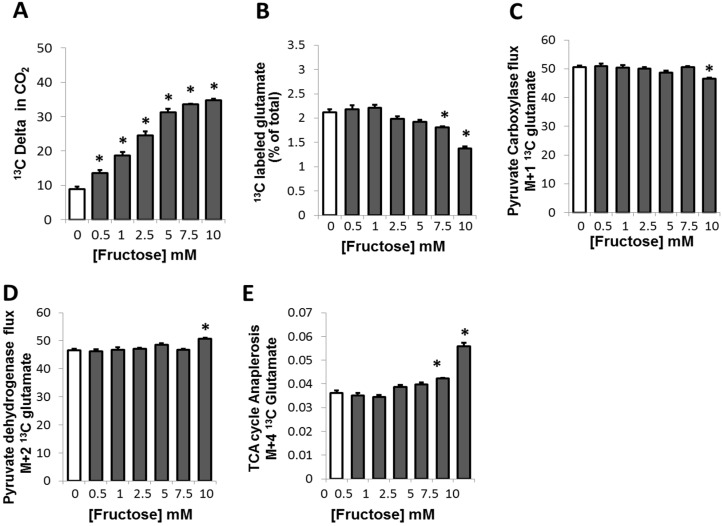
Modulation of ^13^C glucose oxidation and glucose-derived glutamate synthesis via TCA cycle in adipocytes in the presence of fructose (**A**) Oxidation of glucose in adipocytes exposed to fructose in a medium containing a baseline amount of 5 mM of glucose. Oxidation of glucose was measured by the change (delta, Δ) in the ^13^CO_2_ released in the extracellular medium measured as a ratio of ^13^C/^12^C (replacement of ^12^C by ^13^C) in extracellular bi-carbonate; (**B**) Extracellular release of [^13^C]-glutamate in adipocytes exposed to fructose in a medium containing a baseline amount of 5 mM of glucose. ^13^C glucose-derived glutamate release is expressed as a percentage of total glutamate released from glucose; (**C**) Glucose-derived pyruvate entry into TCA cycle via pyruvate carboxylase (PC). PC flux was measured as extracellular [3-^13^C_1_] glutamate using the M_+1_ ΣmC_2_-C_5_ fragment (one ^13^C substitution, *m*/*z* 199 fragment, electron impact ionization) of its ^13^C labeled fraction in adipocytes exposed to fructose; (**D**) Glucose-derived pyruvate entry into TCA cycle via pyruvate dehydrogenase (PDH). PDH flux was measured as extracellular [4,5-^13^C_2_] glutamate using M_+2_ ΣmC_2_-C_5_ fragment (two substitutions, *m*/*z* 200) fragment, electron impact ionization) of its ^13^C labeled fraction in adipocytes; (**E**) TCA cycle anaplerosis in adipocytes by measuring the M_+4_ glutamate flux (M_+4_ ΣmC_2_–C_5_) (four ^13^C substitution, *m*/*z* 202 fragment, electron impact ionization). In each case, ten percent of glucose was supplied as [1,2-^13^C_2_]-d-glucose for a period of 48 h before harvest. * *p* < 0.05 compared to 0 mM of fructose

Oxidation of glucose is the metabolic process by which glucose is catabolized to form CO_2_, and consequently generates ATP for cellular processes. We examined the oxidation of glucose in *in vitro* adipocytes exposed to different concentrations of fructose added to the medium containing a basal level of 5 mM of glucose, as described in the methods section. The oxidation of glucose was examined by measuring the change in ^13^C labeled extracellular CO_2_ released into the medium following treatment of cells with 10% of the total glucose as 1,2 ^13^C_2_ d-glucose tracer. Using the tracer glucose enabled analysis of continuous substrate and product labeling for 48 hours in adipocytes. All concentrations of fructose examined demonstrated a significant dose-dependent increase in the oxidation of glucose shown as a ratio of ^13^C/^12^C (representing the replacement of ^12^C by ^13^C) in extracellular CO_2_ (Figure 1A). These results suggest that fructose augments the catabolism of glucose to CO_2_ to derive more energy for cellular processes in adipocytes in the presence of fructose.

### 2.2. Fructose Altered Glucose Derived-Glutamate Output and Decreased the Cycling of Glucose Carbons through the TCA Cycle

Glutamate formation is an anabolic process resulting from the deamination of α-ketoglutarate, an intermediate of the TCA cycle, and is released into the media from cells when the flux through this cycle is high. Therefore, glutamate can serve as a marker of the cycling activity through the TCA cycle [23]. When adipocytes were exposed to increasing concentrations of fructose, a significant dose-dependent decreasing trend was observed in the ^13^C glutamate formed from labeled glucose (Figure 1B), which reached statistical significance at fructose concentrations of 7.5 mM or higher. A 1.4 fold decrease in ^13^C labeled glutamate was noted in presence of 10 mM of fructose, although the decreasing trend is evident starting at 2.5 mM of fructose. This suggests that the increasing concentrations of fructose correspondingly decrease ^13^C glucose-derived glutamate formation.

Exploring the positions of the ^13^C labelled glutamate in the expelled glutamate in the medium enabled further analysis of flux occurring through the TCA cycle. The M_+1_ (one ^13^C substitution, *m*/*z* 199 (C2–C5)) fragment which indicates lactate and pyruvate cycling and turnover (pyruvate carboxylase (PC) activity) [24] (Figure 1C), demonstrated a decreasing trend with a slight but significant corresponding decrease in flux at the 10mM fructose concentration. On the contrary, the M_+2_ (4,5 labeled ^13^C_2_ glutamate with two ^13^C substitutions, *m*/*z* 200 (C2–C5)) fragment that reflects the pyruvate dehydrogenase activity showed a statistically significant increase only at the highest fructose concentration of 10 mM (Figure 1D). The 2,3,4,5 ^13^C_4_ glutamate (M_+4_, four ^13^C substitutions, *m*/*z* 202 (C2–C5)) fragment reflecting TCA cycle anaplerosis due to the recycling of carbons from 1,2 ^13^C_2_ glucose [25], demonstrated an increasing trend reaching significance at the higher concentrations of fructose, 7.5 and 10 mM (Figure 1E).

Together these results suggest that glucose is not oxidized through the TCA cycle at the lower concentrations of fructose. However, glucose oxidation may occur at high fructose concentrations as demonstrated by the slight increase in PDH/citrate cycling and the recycling of labeled carbons at or greater than 7.5mM fructose concentration.

### 2.3. Presence of Fructose Decreased Glucose-Derived Palmitate Accumulation and Palmitate Release but Augmented Accumulation of Oleate in Adipocytes

**Figure 2:**
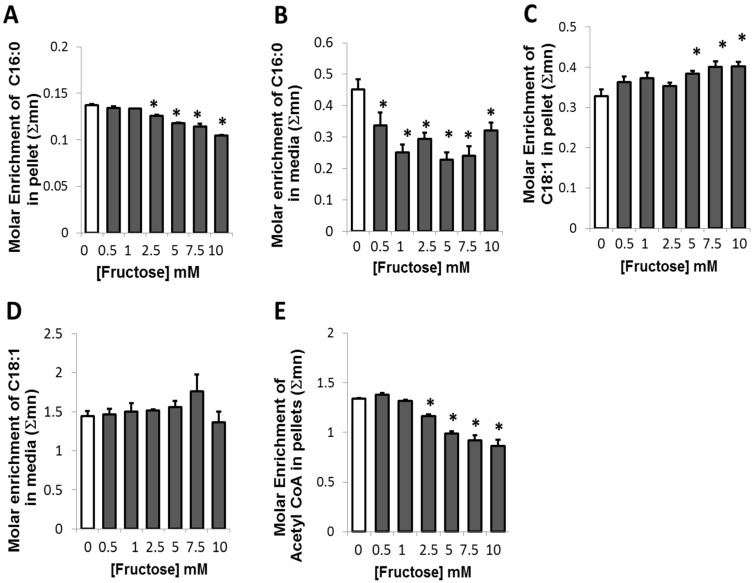
Glucose-derived ^13^C fatty acid and ^13^C acetyl-CoA in adipocytes in the presence of fructose in a medium containing a baseline amount of 5 mM of glucose (**A**) Glucose-derived intracellular ^13^C Palmitate (C16:0) synthesized (**B**) Glucose-derived ^13^C Palmitate (C16:0) released. (**C**) Glucose-derived intracellular ^13^C Oleate (C18:1) synthesized (**D**) Glucose-derived ^13^C Oleate (C18:1) released (**E**) ^13^C glucose-derived acetyl-CoA formed in adipocytes and recovered from palmitate. In each case, ten percent of glucose was supplied as [1,2-^13^C_2_]-d-glucose for a period of 48 h before harvest. * *p* < 0.05 compared to 0 mM of fructose.

Glucose derived pyruvate enters the TCA cycle by the formation of acetyl-CoA and citrate via the PDH reaction (Figure 1D). When citrate is formed in excess, it is expelled from the mitochondria and converted to acetyl CoA, a precursor for fatty acid synthesis. In the presence of fructose, a dose-dependent decreasing trend in the conversion of ^13^C glucose to fatty acid palmitate is observed (Figure 2A) in fructose-treated adipocytes. This decrease was significant at 2.5 mM and higher fructose concentrations. Similarly, a decrease was also observed in the release of the palmitate formed from adipocytes (Figure 2B). The release of palmitate into the medium was decreased at all concentrations of fructose and was not specifically dose-dependent. These results suggest that in the presence of fructose, adipocytes may not utilize much of their glucose for the fatty acid palmitate synthesis or if some amount is indeed formed, it may be converted to the unsaturated fatty acid oleate.

Oleate is a C18:1 unsaturated fatty acid that is formed by the elongation and desaturation of palmitate. While a decrease was seen in the intracellular ^13^C palmitate, a corresponding increase was observed in ^13^C oleate formation in the presence of different concentrations of fructose (Figure 2C). This increase in ^13^C oleate suggested that, in adipocytes, the glucose-derived palmitate formed was preferentially converted to oleate at 5 mM or greater concentrations of fructose. No significant alteration was seen in the release of oleate in the presence of fructose (Figure 2D). Furthermore, in the presence of increasing concentrations of fructose, a significant decrease in the molar enrichment of glucose-derived acetyl-CoA occurred with increasing concentrations of fructose from 2.5 mM of fructose (Figure 2E). Acetyl-CoA is a key precursor for the formation of fatty acids such as palmitate. Hence, the decrease in glucose-derived acetyl-CoA implied that fatty acid synthesis may not be a preferred route for the disposition of glucose in the presence of fructose.

### 2.4. Presence of Fructose Triggered an Increased Conversion of Glucose to Lactate but Decreased the Conversion of Glucose to Glycogen or Ribose

**Figure 3:**
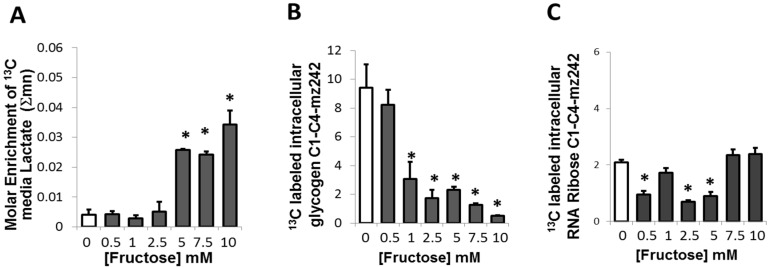
^13^C Glucose-derived media lactate, intracellular glycogen and intracellular ribose in the presence of fructose in a medium containing a baseline amount of 5 mM of glucose (**A**) Media enrichment of ^13^C glucose-derived lactate in adipocytes; (**B**) ^13^C glucose-derived glycogen C_1_–C_4_-mz242 in adipocytes; (**C**) ^13^C glucose-derived intracellular RNA ribose in adipocytes. * *p* < 0.05 compared to 0 mM of fructose

Lactate is an end product of anaerobic glycolysis. It can be formed in cells when there is an excess of glycolysis precursors, such as an increased availability of glucose and other glycolytic precursors including fructose. Lactate production also depends on a competition for pyruvate and NADH between LDH and NADH shuttles (such as malate aspartate and glycerol phosphate) and the pyruvate transporter [26]. In adipocytes treated with different concentrations of fructose added to glucose containing medium, no change in the ^13^C lactate was seen up to 2.5 mM of fructose (Figure 3A). However, more ^13^C glucose was converted to ^13^C lactate in the presence of 5 mM or higher fructose concentration (Figure 3A). This increase corresponds to the increased energy demand for fructose-mediated processes that may be occurring in these adipocytes treated with increasing concentrations of fructose.

Glycogen is a storage form of glucose which is present in adipocytes although at a much lower level than in liver or skeletal muscle [27]. In the presence of fructose, a significant dose-dependent decrease in the intracellular ^13^C labeled glycogen fraction was observed in adipocytes starting at 1 mM of fructose (Figure 3B). These results demonstrated that in the presence of fructose, glucose is not converted to and stored as glycogen, but utilized towards other pathways, particularly increased oxidation by conversion to CO_2_.

Glucose can also be metabolized by oxidative decarboxylation where glucose-derived 6-phosphogluconate yields the 5-C ketose, ribulose-5-phosphate. A decreased conversion of glucose to ribose was observed at fructose concentrations up to 5 mM (Figure 3C). However, 1, 7.5 and 10 mM of fructose did not appear to alter the conversion of glucose to ribose compared to the control, suggesting that this reaction was not robustly altered overall in the presence of fructose.

### 2.5. Presence of Fructose Increases the Flux of Glucose through the SOGC Pathway Resulting in Increased Oxidation of Glucose

**Figure 4:**
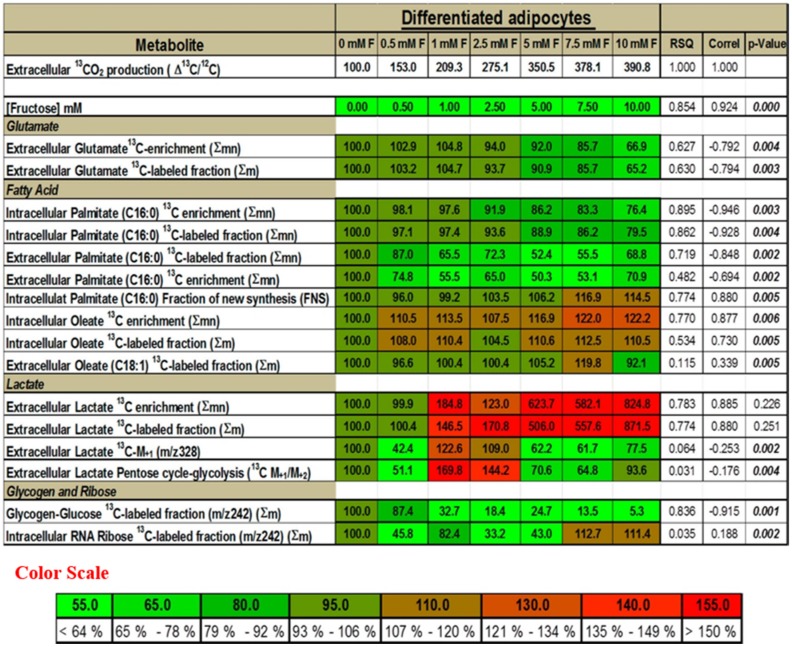
Correlation of the SGBS adipocyte ^13^C isobolome (dependent variables) in response to glucose derived ^13^C delta in CO_2_ (independent reference variable). SGBS adipocyte isobolome (representing the adipocyte ^13^C labeled metabolome)-wide associations in a heat map using the [1,2–^13^C_2_]-d-glucose flux surrogates, are examined for correlation with glucose derived ^13^CO_2_ production across different fructose concentrations. The figure presents regression statistics by the coefficient of determination (RSQ), correlation coefficients (correl, R) and p values for the numeric percent changes among groups (please see Methods) of the adipocyte ^13^C isotopomers and glucose derived ^13^C CO_2_ production. The darkest green in the heat map represents 100% with the lighter green shades representing decreasing % values and the brighter red shades representing increasing % values.

The decreased conversion of glucose to glutamate, palmitate or glycogen in the presence of fructose, but the corresponding increased formation of ^13^CO_2_ from glucose suggested that fructose may be driving glucose through the recently described serine synthesis, one-carbon cycle and glycine cleavage (SOGC) pathway resulting in increased CO_2_ production and ATP synthesis. The SOGC pathway was characterized and described using methotrexate, an inhibitor of the single carbon cycle arm of the SOGC reaction architecture [28]. The characteristic signatures attributable to the SOGC pathway include negative correlation coefficients of ^13^CO_2_ with ^13^C glutamate and with fatty acid palmitate, respectively, which are strong markers of limited TCA cycle flux, turnover and ATP production from glucose. The ^13^CO_2_ formed was negatively correlated with ^13^C glutamate enrichment (*R* = −0.792) and the ^13^C-labeled glutamate fraction (*R* = −0.794), while the fructose dose showed strong positive correlation (*R* = 0.924) with ^13^CO_2_ formed (Figure 4) from glucose. However, similarly to glutamate, the ^13^CO_2_ formed showed negative correlation with intracellular (*R* = −0.946) and extracellular (*R* = −0.694) ^13^C palmitate enrichment and with intracellular (*R* = −0.928) and extracellular (*R* = −848) ^13^C-labeled fraction of palmitate, respectively. The negative correlation of both total ^13^C glutamate and total ^13^C palmitate synthesis with ^13^CO_2_ formed indicated that the flux of the glucose carbons through the TCA cycle was limited and was thus not significantly utilized for anabolic functions such as glutamate and palmitate synthesis. Although the total ^13^C labeled palmitate fraction negatively correlated with ^13^CO_2_ formed, a positive correlation (*R* = 0.880) was observed between the fraction of newly synthesized palmitate (palmitate formed via *de novo* fatty acid synthesis) and ^13^CO_2_. The fraction of newly synthesized palmitate was marginally increased and was likely to be utilized for the synthesis of oleate by the elongation and desaturation of palmitate formed. Intracellular oleate was increased in the presence of increasing doses of fructose (Figure 2C). Another distinguishing signature of this pathway is the significant correlation between glucose-derived ^13^CO_2_ and lactate labeling in response to increasing fructose concentrations (Figure 3A and Figure 4). Total ^13^C lactate enrichment (*R* = 0.885) and ^13^C lactate fraction (*R* =0.880) were increased and positively correlated with ^13^CO_2_ formed in the presence of fructose. The ^13^CO_2_ formed does not come from the pentose cycle as evidenced by the negative correlation between ^13^C M_+1_ lactate (*R* = −0.253) and ^13^C M_+1_/M_+2_ (*R* = −0.176) which would have resulted if glucose were cycled through the pentose phosphate pathway. The negative correlation with glycogen (*R* = −0.915) in addition to palmitate and glutamate synthesis (above) supports the flux of glucose carbons through an alternate pathway. These results suggest that glucose is cycled through the SOGC pathway in the presence of fructose. Glucose carbons are used for the synthesis of serine followed by its conversion to glycine and the subsequent cleavage of glycine to yield increased CO_2_. This alternate pathway produces energy and releases CO_2_ via the one-carbon cycle.

## 3. Discussion

Glucose is an important monosaccharide used by cells as the primary source of energy [10]. It is critical to normal physiological functioning of all cells, particularly in the brain, which normally uses glucose as its sole energy source [11]. The availability of glucose promotes its oxidation and consequent energy production for cells [29].

In adipose tissue and skeletal muscle, glucose is taken up for energy production primarily when food is consumed and circulating glucose is high in the post absorptive state [30]. However the oxidation of glucose can be inhibited under conditions when circulating fatty acids are increased and when glucose and fatty acids are present in excess and compete with each other in metabolic pathways [14,15]. Excess circulating triglycerides can inhibit glucose utilization and glucose metabolism [31]. Nutrients such as fructose also impact glucose utilization as demonstrated in hepatocytes where increased levels of fructose inhibited glucose oxidation and altered the metabolism of glucose [9]. Metabolism of tracer-labeled fructose has been described in rodent adipose tissue [32] and in human adipocytes in our laboratory [33]. However, the impact and influence of fructose on the metabolism of glucose in adipocytes is not known and was examined in this *in vitro* study using tracer labeled 1,2 ^13^C_2_ d-glucose. This study demonstrated that the presence of fructose propelled the utilization of glucose by oxidation which may be occurring via the SOGC pathway and by the conversion of glucose to lactate. However, the flux of glucose carbons decreased in the TCA cycle and for other potential fates including fatty acid synthesis, glycogen synthesis, and ribose synthesis.

The human SGBS pre-adipocyte cell strain was used in this study owing to its high capacity for differentiation to adipocytes [34], its similar metabolic properties to isolated human adipocytes [35]. Adipocytes express the fructose specific transporter, SLC_2_A5 [35] although other specific fructose metabolizing enzyme(s) in adipocytes have not been fully studied. While a very low expressing variant of fructokinase (KHK-A) has been reported in mouse adipose tissue [36], studies by Froesch and Ginsberg in rat adipose tissue did not find any fructokinase activity [32].

In this study, the influence of a range of fructose concentrations mimicking the levels of fructose reported in circulation [37,38] were examined to better understand the effects of fructose on the metabolism of glucose. The basal concentration of fructose in circulation has been reported to be between 0.05 and 2 mM [37,39]. However, serum fructose levels were 17 and 10 mM of fructose in systemic circulation 30 min and 2 h, respectively, following fructose ingestion [37]. Others reported levels of 7.2 and 3.6 mM of fructose at 1 and 2 h following fructose ingestion [39]. The levels of fructose used in this study are similar to fructose levels reported to be attained in plasma from diet. Glucose was present in all conditions at a concentration of 5 mM, representing the normal levels in circulation *in vivo*.

Increasing concentrations of fructose robustly augmented the oxidation of glucose to CO_2_ in a dose-dependent manner indicating an increased demand for energy, in the presence of fructose, resulting in the catabolic process of increased oxidation of glucose in fully differentiated adipocytes. Oxidation of metabolic fuels generates energy in the form of ATP for cellular processes. The response noted in adipocytes is in contrast to that demonstrated by hepatocytes (HepG2 cells) where glucose was oxidized at lower rates in the presence of fructose [9]. The difference in the impact of fructose on glucose oxidation may be attributable to how fructose is metabolized in these two different cell types [40]. Studies in adipocytes [33] have previously demonstrated that labeled fructose is not efficiently oxidized by adipocytes while the fructose carbons are very robustly utilized for anabolic processes such as synthesis of the fatty acid palmitate and glutamate. Hence, it is likely that the energy-requiring anabolic processes triggered by fructose in adipocytes may be supported by augmented oxidation of glucose to provide energy for these processes.

Energy production in cells occurs predominantly via glycolysis and oxidative phosphorylation in the TCA cycle to support cell growth, maintenance, and to perform cellular functions [40,41]. Under aerobic conditions, pyruvate (derived from glucose) may be transported to the mitochondria and converted to acetyl-CoA, a key molecule that stimulates the TCA cycle. The TCA cycle results in complete oxidation of glucose derived acetyl-CoA to CO_2_ and H_2_O concomitantly producing energy by oxidative phosphorylation [42]. The results presented here revealed for the first time that increasing concentrations of fructose dose dependently increased the conversion of 1,2 ^13^C_2_ D-glucose to ^13^CO_2_ by flux through the SOGC pathway. This conclusion is supported by the decreased level of glutamate, a key marker of TCA cycle activity in response to increasing fructose concentrations in adipocytes. The SOGC pathway is an alternate energy-yielding pathway that has recently been described by Tedeschi *et al.* [28] to explain the Warburg effect observed in cancer cells. In this pathway, glycolytic intermediate 3-phosphoglycerate is diverted from glycolysis and utilized for the synthesis of the amino acid serine. Some of the serine synthesized in this manner is converted to glycine in a reaction that is coupled with the one carbon metabolism pathway, yielding ATP. The glycine formed undergoes cleavage to form CO_2_ [28]. The intermediates of the one carbon pathway also contribute to the production of NADPH, a key cofactor that is utilized for fatty acid synthesis. As adipocytes robustly utilize fructose for palmitate synthesis [33], the diversion of glucose through the SOGC pathway in the presence of increasing concentrations of fructose may aid not only in energy production but also in the generation of NADPH precursors needed for fatty acid synthesis. Characteristic hallmarks of the SOGC pathway in adipocytes include (i) the negative correlation of the ^13^CO_2_ produced with the ^13^C labeled glutamate and ^13^C palmitate fractions respectively, which is due to the decreased flux through the TCA cycle (ii) the negative correlation of ^13^CO_2_ with M_+1_ labeled lactate which results from metabolism of glucose through the pentose phosphate pathway (iii) a positive correlation with ^13^C labeled total lactate revealing that in adipocytes treated with fructose, glucose was diverted from the TCA cycle and directed more to the SOGC pathway for oxidation and ATP production. These correlations demonstrated that flux through the TCA and pentose cycle was limited in the presence of increasing concentrations of fructose. The results were lower production of NAD^+^/NADP^+^ and ATP production from glucose oxidation in the mitochondrial energy metabolism pathway. As fructose carbons are not efficiently oxidized in adipocytes [33], the available glucose is primarily utilized via the SOGC pathway which produces energy as well as NADPH for fatty acid synthesis and other anabolic processes resulting from the presence of fructose.

Decreased activity of PC and increased PDH activity as well as increased levels of 2,3,4,5 [^13^C_4_] glutamate resulting from the recycling of carbons via anaplerotic pathways in the TCA cycle were observed only at very high concentrations of fructose. Such alterations, however, were not simultaneously accompanied by an increase in the PDH/citrate cycle and consequent palmitate synthesis from glucose revealing that in the presence of fructose, glucose was not significantly utilized for anabolic functions such as fatty acid or glutamate synthesis. On the other hand, tracer-labeled fructose was robustly used for palmitate synthesis in a dose-dependent manner and was also converted to oleate as noted in our previous study [33]. Although the formation of ^13^C palmitate was not robust and showed a decreasing trend in this study, any glucose tracer-derived ^13^C palmitate that was synthesized from glucose in the presence of fructose was converted to ^13^C oleate. Data demonstrated that oleate accumulated in the adipocytes and was not released in the presence of fructose in these cells. In contrast, both palmitate and oleate were co-released from hepatocytes *in vitro* [9]. *In vivo* studies examining short-term fructose administration showed an increase of about 58% of circulating VLDL triacylglycerols in humans [43]. However, nonesterified fatty acid (NEFA) levels in plasma are thought to be determined by their rate of release from adipose tissue in addition to clearance mechanisms regulating their removal [44]. Intravenous fructose administration decreased serum NEFA levels, due to a reduction in NEFA Ra (plasma NEFA appearance rate) and an increase in the NEFA metabolic clearance rate [44], or left them unchanged [45]. In agreement with such observations, the findings in this study demonstrated that glucose does not contribute to the release of free palmitate and oleate in media in the presence of increasing concentrations of fructose. These results also support an augmented catabolic role for glucose seen with the increased oxidation of glucose (formation of glucose derived ^13^CO_2_) in the presence of increasing concentrations of fructose but a decreased anabolic role as demonstrated by the decreased glutamate and palmitate synthesis in adipocytes from glucose.

**Figure 5:**
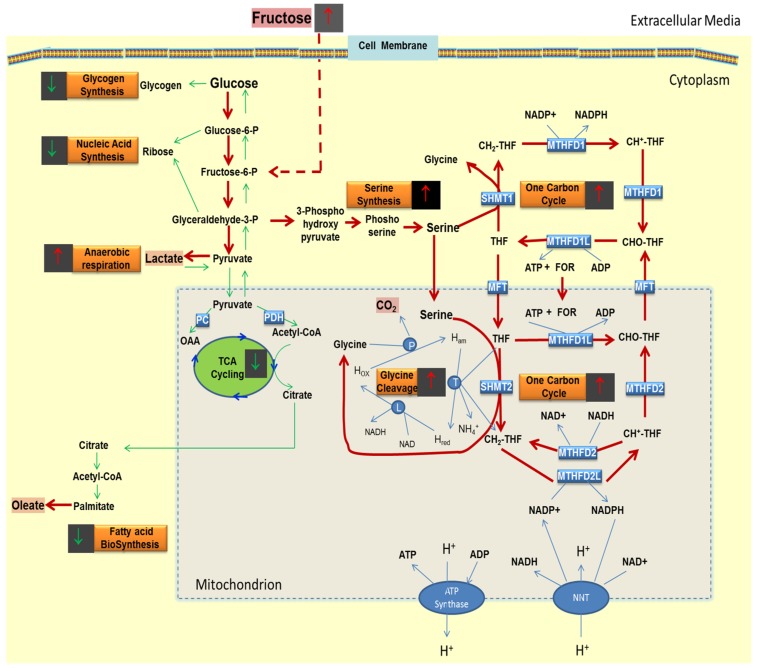
Schematic of the flow of glucose carbons in the presence of added fructose in adipocytes. The red arrows indicate pathways with increased flow of glucose carbons and the green arrows indicate pathways where the flow of glucose carbons are decreased or not significantly altered. PC, pyruvate carboxylase; PDH, pyruvate Dehydrogenase; OAA, oxaloacetate; THF, tetrahydrofolate; SHMT2, serine hydroxymethyltransferase (mitochondria); CH_2_-THF, Methyltetrahydrofolate; CH^+^-THF, 5, 10-methenyl THF; MTHFD2L, methylenetetrahydrofolate dehydrogenase (NADP, mitochondria); MTHFD2, methylenetetrahydrofolate dehydrogenase (NAD) methenyl tetrahydrofolate cyclohydrolase (mitochondria); CHO-THF, 10-formyl THF; MTHFD1L, formyltetrahydrofolate synthetase (mitochondria); MFT, mitochondrial folate transporter (SLC25A32); FOR, formate; MTHFD1, methylenetetrahydrofolate dehydrogenase (NADP) methenyl tetrahydrofolate cyclohydrolase, formyltetrahydrofolate synthetase (cytosol); MTHFD1L, formyltetrahydrofolate synthetase (mitochondria); SHMT1, serine hydroxymethyltransferase (cytosol); NNT, nicotinamide nucleotide transhydrogenase. P-(Glycine cleavage system protein P (glycine dehydrogenase (decarboxylating)); L—Glycine cleavage system protein L (dihydrolipoamide dehydrogenase); T—Glycine cleavage system protein (aminomethyltransferase). The SOGC pathway portion in the figure has been adapted from [28]. This figure does not reflect equilibrium or steady state distributions. It only shows glucose flux relationships by surrogates based on isotopic labeling.

An alternate fate of pyruvate formed from glucose is the conversion of pyruvate to lactate [42]. Lactate production by adipose tissue is dependent on the nutritional state and availability of the precursor glucose [46]. Glucose can be metabolized to lactate in adipose tissue which can provide lactate for hepatic glycogen synthesis. The glucose to lactate conversion can occur to a markedly higher extent in diabetic and obese states [47]. The study reported here revealed that fructose robustly triggered the conversion of glucose to lactate at concentrations of 5 mM and higher resulting in the increased release of lactate from these adipocytes. Although 5 mM of fructose affected glucose to lactate labeling (Figure 3A) and glycogen labeling (Figure 3B), it affected RNA labeling in a non-dose responsive manner. These changes are consistent with molecular crowding stimulated by increasing concentrations of fructose. Specifically, fructose diverted glucose from usual pathways of glucose disposition including fatty acid synthesis, oxidative decarboxylation, or from being deposited into glycogen, and from the cellular pentose to being disposed as lactate. Such responses are consistent with a large and rapid switch as described previously for crowded systems using 1,2 ^13^C_2_-d-glucose as the single tracer [48]. The other fates of glucose-6 phosphate include its conversion to glycogen via glycogen synthesis or its shunting into the pentose phosphate pathway resulting in the production of ribose for nucleotide synthesis. Although adipocytes are primarily the site for lipid storage and contain glycogen only in small amounts, this pathway has been demonstrated in adipocytes although at low levels compared to the liver and skeletal muscle [49,50]. Glycogen is thought to operate in the transition from the fasted to fed state by coordinating glucose and lipid metabolism in adipocytes [27]. Increased glycogen resulting from refeeding may provide a cue to stop lipolysis that is normally active during conditions of fasting and to enable the adipocyte to metabolically reset prior to shifting back to triglyceride synthesis. The increasing concentrations of fructose, which parallels a fed state, dramatically decreased the conversion of glucose to glycogen at all concentrations of fructose. The minor small decreased amount of glucose–derived ribose observed at different concentrations of fructose is consistent with the fact that ribose is not needed in high concentrations in differentiated adipocytes. These data were consistent with the flux of glucose to the SOGC pathway for oxidation in the presence of fructose (Figure 5).

Our overall observation is that fructose alters the metabolism of glucose by driving more glucose through oxidation pathways to acquire sufficient energy to meet the metabolic endpoints of fructose. However, oxidation was not occurring via the oxidative decarboxylation of glucose in the mitochondria, suggesting a potential decrease or impairment of mitochondrial oxidative process in the presence of fructose. Fructose increased lipogenesis with the synthesis of fatty acids including palmitate and oleate as noted in previous studies [33,51]. Excess lipid accumulation is known to be associated with endoplasmic reticulum stress and cellular dysfunction in adipocytes [52]. Thus, decreased mitochondrial oxidative decarboxylation together with excess lipid accumulation through increased lipogenesis of palmitate and oleate [33,51] in the presence of increasing levels of fructose, may lead to adipocyte dysfunction.

Further *in vivo* studies examining the fructose-induced changes in adipose tissue and its systemic effects are warranted to better understand the role of fructose in the pathogenesis of obesity and insulin resistance. While cell culture experiments cannot mimic *in vivo* conditions, they nevertheless can aid in illustrating the changes in pathways that would be difficult to detect at lower concentrations or in *in vivo* experiments. The results of these cell culture experiments showed that fructose profoundly altered intermediary metabolism and impaired glucose metabolism in a competitive manner much the same way as seen in the Randle cycle (11, 12). The Randle cycle is essentially a competition between glucose and fatty acids for oxidation processes in muscle and adipose tissue (12) and hepatocytes (13). This study demonstrated the same effect of fructose in differentiated adipocytes as evidenced by the reduced production of glutamate and fatty acids following fructose treatment. Hence, fructose modified metabolism of glucose in adipocytes in a manner similar to that of fatty acids in liver cells. Utilization of fatty acids has been shown to inhibit the use of glucose directly and without hormonal mediation in isolated heart and skeletal muscle preparations (11). The report here is the first we are aware of that demonstrated competition between these two dietary sugars with increased sensitivity in mature adipocytes. Fructose has the “upper hand” in regulating glucose metabolism: the glucose-fructose cycle is thus a biochemical phenomenon that is determined by fructose availability which controls fuel selection and product disposal via adaptation to the type and amount of sugar and the demand of metabolic processes in adipocytes. Fructose would increase production of intracellular oleate (feast) which could then be used for metabolic needs in times of energy shortage (famine). The mechanisms reported herein add a new chapter to the complexities involved in nutrient and fuel interactions in cells and likely will trigger additional tracer substrate guided investigations in the glucose-fructose interactions field to better understand metabolism of carbohydrate and fat in health and disease processes.

## 4. Experimental Section

### 4.1. Chemicals and Reagents

Dexamethasone, 3-isobutyl-1-methylxanthine, transferrin, cortisol, triiodothyronine (T3), biotin, pantothenate, dichloromethane, trifluoroacetic anhydride, ethanol, potassium hydroxide (KOH), sodium bicarbonate (NaHCO_3_), HCL, acetone, Dulbecco’s Modified Eagle Medium (DMEM) powder media, Hams’ F12 powder media, fructose and glucose were all obtained from SIGMA Chemical Company, (St. Louis, MO, USA). Fetal bovine serum was obtained from Thermo Fisher Scientific (Waltham, MA, USA). Rosiglitazone was obtained from Cayman Chemicals (Ann Arbor, MI, USA); insulin was obtained from Novo Nordisk; [1, 2–1^3^C_2_]-D-glucose (>99% purity, and 99% isotope enrichment for all carbons) was obtained from Cambridge Isotope Laboratories (Andover, MA, USA); methane and helium (>99.99% purity) was obtained from PaxAir (Los Angeles, CA, USA); and n-butanol was obtained from Regis Chemical. Company (Chicago, IL, USA).

### 4.2. SGBS Cell Culture, Fructose Treatment and Labeling Using ([1, 2–1^3^C_2_]-d-Glucose) Tracer

Human Simpson-Golabi-Behmel syndrome (SGBS) preadipocytes, kindly provided by Martin Wabitsch, were used in this study. The cells were cultured as described previously [53] with slight modifications. Briefly, the SGBS preadipocyte cells were cultured at 37 °C in a humidified incubator maintaining a 5% CO_2_ atmosphere. The growth medium consisted of DMEM:F12 (1:1), 33 mM biotin, and 17 mM pantothenate containing 10% fetal bovine serum and 1% penicillin-streptomycin. For this study, SGBS preadipocytes were plated at 2 × 10^5^ cells in 60 mm dishes. The cells were supplemented with 3 mL growth medium (described above) and grown to confluence and induced to differentiate into adipocytes one day post confluence by addition of a serum-free differentiation medium. The differentiation medium consisted of DMEM:F12 (1:1) (obtained by mixing Dulbecco’s Modified Eagle’s Medium without glucose (SIGMA) and Hams F12 nutrient mixture containing 10 mM of glucose (SIGMA) in a 1:1 ratio) to which 25 nM dexamethasone, 500 μM 3-isobutyl-1-methylxanthine, 2 μM rosiglitazone, 0.01 mg/mL human transferrin, 2 × 10^−8^ M insulin, 10^−7^ M cortisol, 0.2 nM T3, 33 mM biotin, and 17 mM pantothenate were added. The cells were maintained in differentiation medium for 4 days after which the medium was changed to a serum-free adipogenic medium consisting of DMEM:F12 (1:1) with 0.01 mg/mL human transferrin, 2 × 10^−8^ M insulin, 10^−7^ M cortisol, 0.2 nM T3, 33 mM biotin, and 17 mM pantothenate. The adipogenic medium was essentially similar to the differentiation medium but without 3-isobutyl-1-methylxanthine (IBMX), dexamethasone and rosiglitazone. The medium was renewed every two days from the initiation of differentiation. SGBS cells completely differentiate into adipocytes by day 14 of differentiation. In order to determine the effects of fructose exposure to adipocytes at concentrations reported in the systemic circulation following exposure to fructose-rich food [37], 0, 0.5, 1.0, 2.5, 5, 7.5, 10 mM of fructose was added to the media at the initiation of differentiation and maintained in the medium until the collection of cells on day 16 of differentiation. In order to trace the metabolism of fructose in adipocytes, and to understand the major flow and fate of glucose-derived carbons in human adipocytes, a labeled glucose tracer ([1,2–1^3^C_2_]-d-glucose) was utilized. In this experimental paradigm, the labeled glucose was used at 10% of the respective glucose concentration in the medium. Under such conditions the ^13^C glucose becomes the single tracer of intermediary metabolism by its positional ^13^C carbon contributions to metabolic products. The tracer was added to cells on day 14 for a period of 48 hours to enable collection on day 16. Cell lysates and media were collected for metabolomic assays on day 16. Each concentration was tested in triplicate cultures. For each replicate, samples from two confluent 60 mM dishes were pooled during collection to ensure sufficient material for all assays. At the time of collection, 6 mL of media was first collected, centrifuged at 2000 RPM for 10 min to remove any dead or floating cells, and then flash frozen. The cells were rinsed twice with cold PBS and aspirated to remove any remaining medium. A 300 μL portion of cold PBS was added to each 60 mm dish, and the cells were scraped and collected, flash frozen and stored at −80 °C until used. Tracer labeled endpoints such as extracellular CO_2_, intra- and extra-cellular glutamine, intra- and extra-cellular fatty acids including palmitate and oleate, lactate, glycogen and ribose, some of which are positionally labeled, were examined as described in the methods and the results are presented below. All media used for the growth, differentiation and maintenance of adipocytes contained a basal amount of 5 mM of glucose, equivalent to the normal blood glucose concentration.

### 4.3. Targeted ^13^C Metabolite Studies and Metabolic Profiling Using ([1, 2–1^3^C_2_]-d-Glucose)] Tracer

Specific extractions were performed as described below, and mass spectral data were obtained using a HP5975N (Agilent, Palo Alto, CA, USA) mass selective detector connected to an HP6890N (Agilent, Palo Alto, CA, USA) gas chromatograph. An Agilent J&W HP-5MS (30 m × 0.25 mm × 0.25 µm; part #: 19091S-433) analytical column was used during glucose, ribose, glutamate, and lactate analyses. For analysis of fatty acids and CO_2_, an Agilent J&W Scientific DB-23 (60 m × 0.25 mm × 0.15 µm; part #: 122–2361) column was used. Helium (>99.99% purity) was used as the carrier gas in the electron impact ionization (EI) mode and methane (>99.99% purity, PaxAir, Los Angeles, CA, USA) in the chemical ionization (CI) mode. Sample injections were performed using 100 split ratios directly into the heated (250 °C) and pressurized inlet interfaces. Results of mass isotopomers in glutamate are reported as molar fractions where m1, m2, *etc.* indicate the number of ^13^C atoms in the molecule [54]. The enrichment, Σmn, is the weighted sum of the labeled species (Σmn = m1X1 + m2X2 + m3X3…) [55,56]. Lactate was extracted from cell culture media (0.2 mL) by ethylene chloride after acidification with HCl, derivatized to its propylamine-heptafluorobutyrate ester form and applied to the column [57]. Enrichment of ^13^C-labeled acetyl units derived from [1,2–1^3^C_2_]-d-glucose in palmitate and oleate reflect *de novo* fatty acid synthesis, elongation, and desaturation, as determined by mass isotopomer distribution analysis (MIDA) [57,58]. Natural ^13^C abundance of the target and derivatizing compounds are routinely extracted for MIDA calculations [59]. For analysis of fatty acid synthesis, palmitate, and oleate from media or cell pellets were saponified with 30% KOH and 100% ethanol and extracted using petroleum ether. Fatty acids were converted to their methylated derivative using 0.5 N HCl in methanol, and methyl palmitate and methyl oleate were dried, reconstituted in hexane and injected into the GC-MS and monitored at *m*/*z* 270, and *m*/*z* 264, respectively. Analyses of complete oxidation of [1,2–1^3^C_2_]-d-glucose to ^13^CO_2_, were performed using 100 µL media samples in GC vials. To each sample 50 µL of 0.1 M NaHCO_3_ was added, followed by 50 µL of 1.0 M HCl. The vials were immediately capped and placed on the GC/MS sample tray for analysis of the *m*/*z* 44 and *m*/*z* 45 ion group [60]. Only ^13^C labeled product fractions, after their parent ion concentrations are resolved via standard Chemstation Integrator diagnostics following background subtraction, are reported. The internal standard is the ^13^C labeled glucose and its products as it gets metabolized following its addition on day 14 in adipocytes for a period of 48 h as described above. Glutamate was extracted from 100 µL media using 100 µL ultrapure water and 100 µL HPLC grade acetone. The mixture was frozen at −80 °C for one hour, vortexed for 60 s and centrifuged at 13,000 rpm for 30 min. The supernatant was transferred into a 13-inch glass test tube on ice and dried under nitrogen. Glutamate was converted to its N-trifluoroacetyl-n-butyl (TAB) derivative in 2 steps [25]. Samples were mixed with 200 µL 3.0 M HCl in n-butanol and incubated at 100 °C for 1 h to form butyl esters. Samples were treated next with 100 µL dichloromethane and 25 µL trifluoroacetic anhydride at room temperature for 20 min to form TAB-glutamate, and the products were dried under nitrogen. The residues were transferred into GC vials in 200 µL dichloromethane. When [1,2–1^3^C_2_]-d-glucose is processed by glycolysis followed by pyruvate dehydrogenase, the [1,2–1^3^C_2_]-acetyl-CoA produced is used to form [4,5–1^3^C_2_]-glutamate. Alternatively, [2,3–1^3^C_2_]-glutamate is produced from [1,2–1^3^C_2_]-d-glucose when it is processed via the pyruvate carboxylase pathway, which leads to [2,3–1^3^C_2_]-oxaloacetate. Under electron impact ionization (EI) conditions, ionization of TAB-glutamate produces two groups of fragments, *m*/*z* 198→202 and *m*/*z* 152→155, corresponding to the C_2_–C_5_ and C_2_–C4 fragments of glutamate [61]. Therefore, detection of the ^13^C label on C4 and C_5_ of the m/z 200 M_+2_ glutamate fragment ion reflects pyruvate dehydrogenase activity and hence oxidative phosphorylation (catabolic use of glucose). Pyruvate carboxylase yields new (net) oxaloacetate for the cycle from fructose, which forms the anabolic (anaplerotic) substrate for the cycle. [1,2–1^3^C_2_]-acetyl-CoA enrichment is calculated based on palmitate M_+4_/M_+2_ ratios as described previously [62]. Flux surrogates are used throughout data interpretations, instead of flux calculations, whereby not only metabolite concentrations but their turnover rates are depicted by specific (positional) ^13^C labeling and their associations with fructose dosing. Additional tracer uptake and disposal mechanisms are shown in Figure 4 as the function of tracer substrate uptake (^13^C labeled fraction). This method of data presentation is consistent with and follows biotechnology roadmap standards using ^13^C labeling, correction for naturally occurring isotopes, dynamic labeling (changes in positional labeling) based on fructose dosing and metabolite concentrations as surrogate markers of flux [22].

### 4.4. Quantitative Metabolite Analysis

Media and cell pellet metabolic products were quantified and compared by integrated peak area values under corresponding total ion current (TIC) ID logs as determined by retention times in the selected ion monitoring mode by blinded spectra processing personnel (see acknowledgments). Average peak width values were less than 5 s (ChemStation Integrator, Agilent, Palo Alto, CA, USA) using identical injection volume, solvent strength and split ratios with that of natural ^13^C-labeled external chromatographic standards. Concentration-dependent integrated chromatographic TIC areas are displayed as arbitrary values of metabolite concentration among the control and fructose treated groups.

### 4.5. Serine Oxidation Glycine Cleavage (SOGC) Pathway Analyses

Rapid system-wide association study (SWAS) evaluation of SGBS cells was performed by the color assisted visual isotopolome data matrix screening tool [21,63] to diagnose phenotypic serine oxidation and glycine cleavage as markers of glucose-deriving substrate level phosphorylation (ATP synthesis) [28] and its response to fructose treatment. The SOGC pathway is routinely measured by the positive correlation between ^13^CO_2_ release and ^13^C lactate labeling, at the expense of ATP synthesis in mitochondria via glutamate (TCA cycle) and fatty acid labeling (citrate shuttling) as the central CO_2_ releasing mechanism from glucose.

### 4.6. Statistical Analysis

Data in Figure 1, Figure 2 and Figure 3 are presented as arithmetic mean (average) plus standard error (SEM) of three independent observations using three independent integration results with background subtraction of the natural labeled ^13^C standard and its derivatizing agent. ANOVA with Dunnett’s test was used, and *p* ≤ 0.05 was considered to indicate statistically significant differences in metabolite flux results in response to fructose treatment of adipocytes. For evaluating the level of difference among groups, the percentages of changes from 0 mM F were calculated and a linear regression analysis with RSQ (coefficient of determination) measurement was performed to describe the relationship between the SGBS adipocyte ^13^C isobolome (dependent variables) and glucose derived ^13^C delta in CO_2_ at different fructose concentrations. The “p” values in the SGBS-isobolome ([1,2–^13^C_2_]-d-glucose; Figure 4) are calculated based on the percent numeric values that are used for the correlation statistic and they do not determine significance for correlation strength, which is described by coefficients. The p values signify how different the percent values are from each other, as groups, among variables. As can be noted, ^13^CO_2_ and ^13^C lactate percent changes are in closer numeric ranges (hundreds), than other variables, thus the high p values indicate that these are responsive markers of the SOGC pathway [21]. High correlation coefficients and close numeric ranges (no significant differences in the percent ranges among ^13^CO_2_ release and changes in lactate release) are signs of close variable response after fructose treatment, consistent with the SOGC pathway.
